# Male mating and female postmating performances in cotton mealybug (Hemiptera: Pseudococcidae): effects of female density

**DOI:** 10.1093/jee/toz030

**Published:** 2019-02-23

**Authors:** Haojie Tong, Zihao Li, Wanyi Ye, Ying Wang, Mohamed Abdelwanees Abdelmowla Omar, Yan Ao, Fei Li, Mingxing Jiang

**Affiliations:** Institute of Insect Sciences, Zhejiang University, Hangzhou, China

**Keywords:** cotton mealybug, mating behavior, reproduction, life trade-off, biological invasion

## Abstract

For insects, female density is closely related to reproductive output. However, little is known about the effects of female density on male mating and female postmating performances. Here, we explored the effects of female density in cotton mealybug, *Phenacoccus solenopsis* Tinsley (Hemiptera: Pseudococcidae), an invasive, rapidly spreading pest in Asia damaging multiple crops and horticultural plants. Using USB digital microscopes, we investigated the frequency, duration, and intervals of mating for males that were individually supplied with 1, 5, 10, and 15 females. We also evaluated the reproduction of mated females and the sex ratio of their offspring. As the female density increased, males mated with more females while substantially shortening mating intervals. Mating occurred actively at the densities of 10 and 15 females, where males mated four times on average, and some mated 6–9 times. However, mating duration and the observed reproductive parameters of females (preoviposition period, overall period from formation of ovisacs to female death, fecundity, and offspring sex ratio) did not differ significantly with female density. A weak trade-off existed between males’ mating frequency and longevity, but there was no relationship between females’ fecundity and longevity. In conclusion, despite their short lifespan, *P. solenopsis* males have a high mating capacity, and their mating frequency and intervals can be significantly affected by female density. In contrast, female density has little influence on females’ postmating performance. Our findings indicate the significance of the reproductive biology and life history strategies for rapid establishment and population development of mealybugs in newly invaded regions.

In insects, mating performance of each sex is associated with various factors such as mating/reproductive history (Uhia and Rivera 2005, Kant et al. 2012, Colares et al. 2015), age (Kawazu et al. 2014, Liu et al. 2014, Ekanayake et al. 2017), nutritional condition (Aluja et al. 2009), body size/mass (Aluja et al. 2009, De Luca 2015, Ekanayake et al. 2017), and population density (Shelly et al. 2013). When the relative density of mates changes, females or males may have different mate-encountering rates, searching costs, or mate-choosing opportunities, ultimately leading to changes in mating performance and reproductive outcomes (Kokko and Rankin 2006, Lehmann 2012). Therefore, mate density is a major factor in insects’ reproductive biology and population dynamic mechanisms.

The effects of male density on female or male mating performances have been well documented for a number of insect species, e.g., water strider (Sih and Krupa 1995, Lauer et al. 1996), weevil (Flay et al. 2014), field cricket (Atwell and Wagner 2014), butterfly (Holveck et al. 2015), and damselfly (Gomez-Llano et al. 2018). However, little is known about the effects of female density, which have been studied only in a few insects. In the salt marsh-inhabiting planthopper *Prokelisia dolus* Wilson (Hemiptera: Delphacidae), female density was found to differentially influence males’ ability to locate mates and male reproductive potential (Langellotto and Denno 2001). In the water strider *Aquarius remigis* (Say) (Hemiptera: Gerridae), however, no relationship between female density and male mating frequency was detected (Lauer et al. 1996). Da Silva et al. (2013) observed the mating frequency and mating duration of the citrus mealybug *Planococcus citri* Risso (Hemiptera: Pseudococcidae) and citrophilus mealybug *Pseudococcus calceolariae* Maskell (Hemiptera: Pseudococcidae) at different female densities (1, 2, 4, 8, and 16 females per male). Both mealybugs showed increased mating frequency as the female density increased. Moreover, *P. calceolariae* males mated significantly faster at the highest female density (i.e., 16 females) than at most of the other densities. The *P. citri* males also mated fastest at the highest female density, but the difference was not statistically significant. Because insects are highly diverse and different species may have evolved specific life history strategies (Bernays 2009), the responses to female density probably differ among species. Therefore, this topic needs to be studied in a range of insect species.

For females, their postmating performance, e.g., reproductive outputs and survivorship, as well as the traits of their offspring, are affected by several aspects of their mates or themselves. Female fecundity is partially determined by mating history and by the age of available males (Kant et al. 2012, Kawazu et al. 2014, Tobin et al. 2014, Colares et al. 2015, Wang et al. 2018). Specifically, females have a higher reproductive output if mated with virgin males (Torres-Vila and Jennions 2005) or experienced multiple matings (Kant et al. 2012, Wang et al. 2018). Mating can affect females’ longevity positively (Villarreal et al. 2018), or negatively (Yu et al. 2014), through the delivery of male-derived substances, e.g., male accessory gland and seminal vesicle proteins, via ejaculates. The mating history of females can also affect several traits of their offspring, including the sex ratio (Kant et al. 2012), survival rate (Ivy and Sakaluk 2005), body size, and dispersal ability (Gottlieb et al. 2014). In short, life parameters of mated females and their progeny could be indicative of the nature of mating experienced by the females, and thus should be included in analyses of associations between male mating performance and female density.

The solenopsis mealybug, *Phenacoccus solenopsis* Tinsley (Hemiptera: Pseudococcidae), is native to North America, and is an invasive alien pest in Asia and beyond (Wang et al. 2010). It can rapidly establish and develop population in newly invaded regions, and is continually extending its geographic range because of its adaptive life features, including its broad host range, high fecundity, protective benefits from mutualistic ants, and its ability to adapt to diverse abiotic environmental conditions (reviewed by Tong et al. 2018). Both adults and crawlers damage plants by sucking cell sap from phloem tissue of leaves, stems, twigs, flower buds, and young bolls, causing premature leaf drop, dieback, and even the death of whole plant. Besides, honeydew excreted by these insects serves as a medium for the growth of sooty mold that hinders the photosynthetic ability of plants (Saeed et al. 2007). In some Asian countries, particularly India and Pakistan, cotton mealybug has caused serious economic losses to cotton production (Hodgson et al. 2008, Nagrare et al. 2009). In China, this mealybug was ranked as one of the 21 most important invasive insects to be managed with priority (Ministry of Agriculture 2013).

Like other mealybug species, *P. solenopsis* exhibits typical sexual dimorphism. Females have three instars during the nymphal stage, and their adults are similar to nymphs morphologically but their body size increases by several times during feeding. Males proceed to the pupal stage after the second nymphal instar. Adult males have wings and a small body size, and do not feed (Zhu et al. 2011). Strikingly, female adults are sedentary, highly aggregative, and have a long lifespan (0.5–2 mo, depending on hosts and temperatures) (Prasad et al. 2012, Zhao et al. 2016), while male adults survive for only 2–4 d (Zhu et al. 2011). In the past decade, the mating and reproductive features of *P. solenopsis* have attracted much research interest, including its mating frequency (Wei et al. 2010), mating duration (Wei et al. 2010, Zhang et al. 2015), mating preferences (Xu et al. 2016), fecundity (Wang et al. 2014), and associations between mating and females’ ovarian development and oviposition (Huang et al. 2013, Zhang et al. 2015, Arif et al. 2016). Wei et al. (2010) reported that males of cotton mealybug mated only once during their lifetime, but an average of 4.5 matings was reported by Wang et al. (2014). The mating history of males had no effect on the production of progeny (Huang et al. 2013).

Despite the studies mentioned earlier, little is known about the male mating responses to female density in *P. solenopsis*. In natural populations, female adults are expected to have a higher density than male adults during certain seasons, because females live much longer than males. Thus, each living male would have the chance to mate with several females, as observed by Wang et al. (2014). Considering that the short-living males might shift their mating performances to achieve the largest reproductive output, it is of great value to investigate their responses to female density, including mating frequency, mating interval, and mating duration.

Here, using USB digital microscopes, we observed the mating frequency, mating duration, and mating interval of *P. solenopsis* males that were supplied with different numbers of females (1, 5, 10, and 15 females per male). We also recorded the preoviposition period, merged ovipositional and postovipositional period, and fecundity of mated females in each group, and the sex ratio of their offsprings. The relationships between males’ longevity and their mating frequency, and between females’ longevity and their reproductivity, were analyzed. Our aims were to clarify the male mating responses to female density and their reproductive significance for this mealybug, and to improve our understanding of the importance of reproductive biology for population development in insects, particularly mealybugs.

## Materials and Methods

### Insects and Host Plants

*P. solenopsis* used in this study were derived from a colony that was established from individuals collected from the ornamental plant, Rose of Sharon, *Hibiscus syriacus* L. (Malvales: Malvaceae) in Jinhua, central Zhejiang, China, in June 2016. This colony was reared on tomato plants in a climatically controlled chamber maintained at 27 ± 1°C and 75% relative humidity (RH) with a photoperiod of 14:10 (L:D) h. Two-day-old virgin female adults and newly emerged male adults, both of which were sexually mature (Zhang et al. 2015), were used for experiments.

Tomato plants (cv. Hezuo-903, Shanghai Changzhong Seeds Industry Co., Ltd, China) were grown in an insect-free greenhouse (Zijingang Campus, Zhejiang University) that was maintained at 26–28°C under a natural photoperiod, supplemented with electric lighting when necessary. Plants were cultivated individually in plastic pots (10 cm in height, 12 cm in diameter). When plants grew to the 8–10 branches stage, branches and leaves were used for rearing mealybugs.

### Effects of Female Density on Male Mating Performance

In this experiment, one newly emerged male was separately supplied with 1, 5, 10, and 15 virgin, sexually mature females (four female-density treatments in total). In each treatment, females were first transferred carefully onto one fresh tomato leaf at the bottom of a Petri dish (1.5 cm in height, 6 cm in diameter), and then one male was released into the dish. Immediately, male mating performance was recorded under a USB digital microscope (Mustech Electronics Co., Ltd, Shenzhen, China) positioned over the dish. The recorded data were stored on a laptop computer connected to the microscope. In total, 16–36 males were observed in each treatment, with each male serving as one replicate (Table 1). For each male, the recording continued until it died.

**Table 1.** Mating performance of *P. solenopsis* males in different female density treatments

Female density treatments	No. males observed (no. mated males)	Mating frequency (range)	Mating duration (range; s)	Mating intervals (range; h)
1F:1M	36 (19)	0.5 ± 0.1 (0−1)a	290.4 ± 39.7 (71−652)a	29.2 ± 8.5 (0.1−66.7)a
5F:1M	16 (15)	2.1 ± 0.2 (0−3)b	212.1 ± 13.5 (132−567)a	15.4 ± 2.7 (3.4−44.1)ab
10F:1M	16 (16)	3.7 ± 0.3 (2−5)c	247.6 ± 23.2 (162−413)a	7.3 ± 1.6 (0.1−18.3)bc
15F:1M	24 (24)	4.0 ± 0.5 (1−9)c	238.3 ± 18.3 (103−408)a	3.7 ± 1.2 (0.1−16.9)c

Values (mean ± SE) within the same column followed by the same letter are not significantly different at *P* = 0.05 (Tukey’s HSD test).

The above observations were performed in the laboratory at 25.2 ± 0.2°C, with illumination provided by fluorescent tubes and natural light from 8:30 a.m. to 10:30 p.m. and by one table lamp from 10:30 p.m. to 8:30 a.m. The leaves in the dishes were replaced with fresh ones twice every day. To facilitate recording, we selected flat leaves to minimize the space between the leaf and the bottom of the dish, so that mealybugs were restricted to the upper leaf surface.

Based on the data collected, we counted or calculated the number of matings (mating frequency), mating duration (i.e., duration of copulation), mating interval (duration from the end of one mating to the start of the next mating), and longevity for each tested male. The proportion of mated males in each treatment was calculated.

### Effects of Female Density on Reproductive Performance of Females

To obtain mated females, we manually observed the mating process of females at four random times during 8:30 a.m. to 10:30 p.m. in the above experiment. Each mated female was marked with a small, black spot on the dorsal side of its thorax with a marker pen (this marking process did not affect male mating performance; data not shown). As males died, 15–22 marked females were randomly sampled from each female-density treatment, and used for observation of their reproductive performance. Each female served as one replicate.

The sampled females were individually reared in Petri dishes (1.5 cm in height, 9 cm in diameter), and supplied with a tomato branch that was wrapped at the base with moistened cotton. The dishes were placed in a chamber controlled at 27 ± 1°C with a photoperiod of 14:10 (L:D) h, and 75% RH. For each female, we recorded the date when its first ovisac formed, the date when it died, and the number of offspring. The crawlers were reared in the dishes until they grew to pupae (males) or third-instar nymphs (females), when their sex was determined. The branches inside the dishes were replaced with fresh ones every 2 d during these experiments. For each observed female, we calculated the preovipositional period (from adult emergence to ovisac formation), the merged ovipositional and postovipositional period (from first ovisac formation to female death), fecundity, and the sex ratio of its offspring. We could not separate the ovipositional period and postovipositional periods, because females normally stayed with the ovisacs, which made it difficult to judge the end date of reproduction accurately.

### Statistical Analysis

Analysis of variance (ANOVA) was performed to assess the effects of female density on observed life features. Prior to ANOVA, data were tested for normal distribution and variance homogeneity using Kolmogorov–Smirnov’s and Levene’s tests, respectively. To meet these requirements, male mating interval and preovipositional period data were log-transformed; data for merged ovipositional and postovipositional period and fecundity were square-root transformed; and sex ratio data were arcsine square-root transformed. Means were compared using Tukey’s honest significant difference (HSD) posthoc tests. Linear regression analyses were conducted to detect relationships between male longevity and mating frequency, and between female longevity and fecundity, by pooling the data from all treatments. All analyses were performed using the statistical software system SPSS v.20 (IBM Statistics, SPSS 2011).

## Results

### Effects of Female Density on Male Mating Performance

**Fig. 1.**
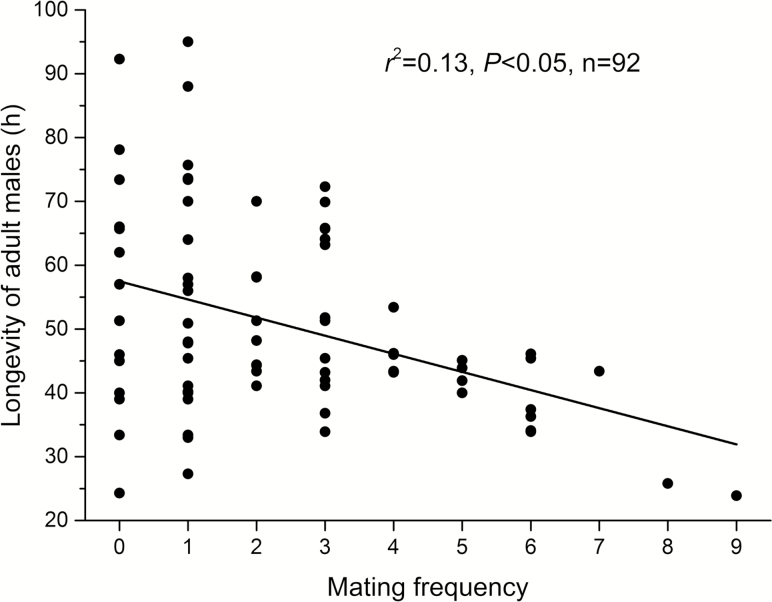
Linear regression (fitting line) between *P. solenopsis* males’ longevity and their mating frequency. Each circle represents one male.

In each of the four female-density treatments, we observed the mating performance of at least 16 males. The ANOVA showed that female density (number of females available to the male) significantly affected the mating frequency of males (*F* = 37.056; df = 3, 84; *P* < 0.001). When supplied with one female, only 52.8% of males mated, and each of them mated only once throughout its life. When five females were available, 93.4% of males mated, and they mated twice on average. In both the 10- and 15-female treatments, all males mated and the average number of matings was 3.7 and 4.0, respectively, which did not differ significantly (Table 1). Notably, some males supplied with 15 females mated 6–9 times (Table 1, Fig. 1). These results revealed that, as the number of females increased, males gradually increased their mating activity, reaching a peak (four matings on average) at the density of around 10 females.

The mating duration of males was longer in the one-female treatment (290 s per mating on average) than in the other treatments (212–238 s). In addition, the mating duration varied more in the one-female treatment than in the other treatments (Table 1), but this difference was not statistically significant (*F* = 1.372; df = 3, 65; *P* = 0.259).

The mating interval varied significantly with female density (*F* = 10.423; df = 3, 44; *P* < 0.001). The more females supplied, the shorter the mating interval (Table 1). There was a significant negative linear relationship between male longevity and mating frequency (Fig. 1).

### Effects of Female Density on Reproductive Performance of Females

**Fig. 2.**
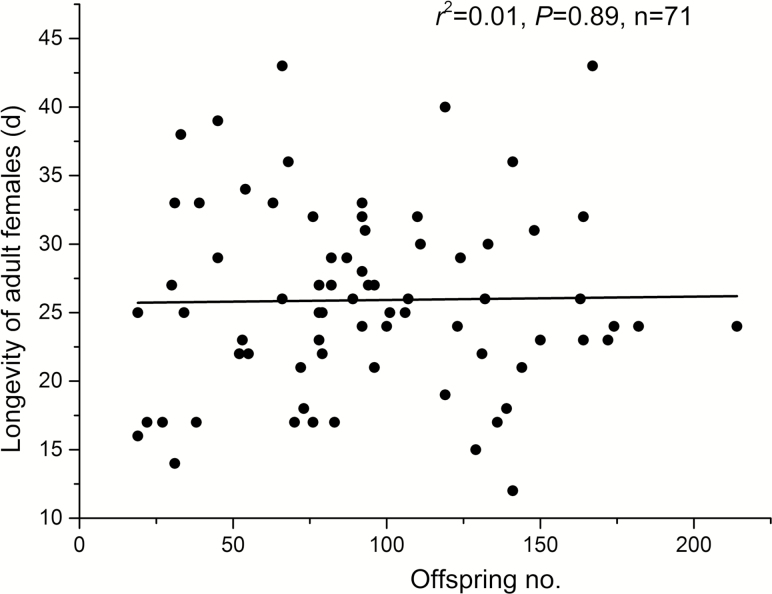
Linear regression (fitting line) between *P. solenopsis* females’ longevity and their fecundity (number of offspring). Each circle represents one reproductive female.

None of the reproductive parameters observed in females was significantly affected by female density, including the preoviposition period (*F* = 0.422; df = 3, 69; *P* = 0.738), merged ovipositional and postovipositional period (*F* = 1.306; df = 3, 69; *P* = 0.279), fecundity (*F* = 0.613; df = 3, 69; *P* = 0.609), and sex ratio of their offspring (*F* = 0.307; df = 3, 69; *P* = 0.820) (Table 2). There was no significant association between the longevity and fecundity of females (Fig. 2).

**Table 2.** Reproductive performance of mated *P. solenopsis* females and sex ratios of their offspring in different female density treatments

Female density treatments	No. females observed	Preovipositional period (range; d)	Ovipositional and postovipositional period (range; d)	Fecundity (range)	Sex ratio^*a*^ of offsprings (range; %)
1F:1M	15	11.3 ± 1.0 (5−21)a	13.8 ± 1.8 (3−25)a	98.0 ± 12.9 (30−182)a	50.0 ± 6.1 (11.7−93.0)a
5F:1M	22	11.8 ± 0.6 (7−20)a	16.1 ± 1.4 (5−30)a	99.4 ± 8.7 (38−148)a	47.7 ± 4.6 (13.3−79.8)a
10F:1M	20	12.6 ± 1.0 (5−28)a	12.4 ± 1.4 (3−24)a	82.4 ± 9.8 (19−164)a	44.7 ± 4.3 (14.3−80.0)a
15F:1M	16	12.3 ± 1.0 (6−25)a	13.1 ± 1.4 (5−27)a	97.5 ± 12.1 (27−182)a	42.7 ± 6.3 (11.1−93.0)a

Values (mean ± SE) within the same column followed by the same letter are not significantly different at *P* = 0.05 (Tukey’s HSD test).

^*a*^Proportion of males.

## Discussion

Here, using USB microscopes, we recorded for the first time the detailed mating responses of *P. solenopsis* males to female density. As the female density increased, males increased their mating frequency and shortened their mating intervals; however, the mating duration did not change significantly. Surprisingly, female density had little effect on postmating reproductive performance. As we predicted, the longevity of males decreased as their mating frequency increased, but the longevity of females was not related to their fecundity.

Close relationships between female density and male mating performance have been observed in a few insect species, as described previously. In mealybugs, such as *P. citri*, *P. calceolariae* (Da Silva et al. 2013), and *P. solenopsis* (this study), each male is able to mate with several females. Some *P. solenopsis* males mated 6–9 times during their lifetime (Table 1, Fig. 1). This finding, together with the observed short mating intervals (Table 1), suggests that this mealybug has a high mating capacity. Such a feature may be a factor in its adaptive nature. First, because *P. solenopsis* females are highly aggregative (Huang et al. 2012), males will frequently find females at a high density and successfully mate with a number of females. Second, because males are short lived (Vennila et al. 2010, Huang et al. 2012) and lack a strong flight capacity (Wei et al. 2010), a high mating capacity would allow them to mate frequently and rapidly before death, thereby maximizing their reproductive output and promoting population development. Thus, these mating features can help this mealybug overcome the shortcomings of its life history, e.g., the short lifespan and weak flight capacity of males, the sedentary lifestyle, and the lack of wings in females.

Another important finding was that *P. solenopsis* female density did not significantly affect male mating duration or female reproductive performance (Tables 1 and 2). Moreover, males’ mating experience appeared to have little effect on mating duration (otherwise the value would vary more widely than that observed among the density treatments where males mated multiple times). This result differs from those reported for other insects (Liu et al. 2010, Omkar and Singh 2010, Omkar et al. 2013, Wang et al. 2016, Wu et al. 2018). Thus, we argue that *P. solenopsis* males might have evolved a sperm investment strategy that meets females’ reproductive requirements: once meeting a suitable female, they would transfer a ‘definite’ amount of sperms to the female, and the females, once mated, would produce a ‘definite’ amount of offspring, irrespective of their density in the population. This strategy may decrease the risk of sperm loss caused by the death of mated female, and contribute to the population development during their invasion. Further work is required to learn whether the sperm investment strategy of *P. solenopsis* males will be affected by their age.

We found a medium level of mate choice in *P. solenopsis*, as observed in the one-female treatment where approximately half (52.8%) of tested males mated. This mating rate was similar to that reported for *P. citri*, where 50% of males mated with single paired females (Da Silva et al. 2013). In *P. calceolariae*, however, a higher mating rate, 88%, was detected for males in pair settings (Da Silva et al. 2013). Overall, mealybugs do not have a high mate choice, that is, *P. solenopsis* males tend to mate with females once they meet rather than waiting for the most suitable ones during their short lifespan. In terms of biological invasion, this is much favorable for establishment in newly invaded regions, where both female and male mates are normally scarce.

Most females mated only once in all treatments; however, there were one and two females that mated twice in the 5- and 15-female treatments, respectively. Thus, females could mate multiple times in their lifetime, especially when sufficient males are available. The same result has been reported previously for cotton mealybug (Wei et al. 2010), and for a number of other mealybug species, such as the long-tailed mealybug *Pseudococcus longispinus* (Targioni Tozzetti) (Hemiptera: Pseudococcidae), obscure mealybug *Pseudococcus viburni* (Signoret) (Hemiptera: Pseudococcidae), vine mealybug *Planococcus ficus* (Signoret) (Hemiptera: Pseudococcidae) (Waterworth et al. 2011), and the grape mealybug *Pseudococcus maritimus* (Ehrhorn) (Hemiptera: Pseudococcidae) (Waterworth and Millar 2012). Multiply mating (with same and/or different males), by contrast to single mating, would favor *P. solenopsis* females to enhance their reproductive success. Further research is needed to investigate the mating and reproductive outputs of *P. solenopsis* females maintained at different densities while supplied continuously with male(s).

In insects, there may be a trade-off between mating and survival, as their longevity tends to decrease as the mating frequency increases (Oliver and Cordero 2009, Metzler et al. 2016). This trade-off may not exist for some insects (Camacho-Garcia et al. 2018). It exists in *P. solenopsis* males, as revealed by the significantly negative relationship between males’ mating frequency and their longevity (Fig. 1). Notably, the males that mated 0–3 times did not differ significantly in their lifespan (Fig. 1), suggesting that such a trade-off is not strong. This is consistent with our speculation that males of cotton mealybug have a high mating capacity. To gain such a capacity, males (which do not feed during their lifetime, and have a low flight capacity) probably allocate a large proportion of energy to reproduction and little energy to flight or other behaviors.

Insect lifespan and reproduction are strongly associated with nutrition, in that the ratio and amount of nutrients consumed can affect life expectancy and reproductive investment (Malod et al. 2017). However, such a nutrient-related effect does not exist in *P. solenopsis* females (Fig. 2). This could be partially explained by the fact that *P. solenopsis* females do not need to spend too much energy on flight, movement, defense against natural enemies, or other nonreproductive performances, because they are wingless, sedentary, covered with wax (Zhu et al. 2011), and have a mutualistic relationship with ants (Zhou et al. 2014). Therefore, most energy could be allocated to reproduction and the maintenance of the somatic body.

In conclusion, we discovered a number of mating and postmating reproductive performances in *P. solenopsis* with potential adaptative significance during its life history. This is one of the few studies on cotton mealybugs that has addressed mating capacity, reproductive outcome, trade-offs, and their associations with female density. Our findings help to explain why *P. solenopsis* can establish and increase its population size so rapidly after arriving at new regions (Huang et al. 2011). The results may also be useful for further research on reproductive biology in other short-lived insect species.
